# Factors affecting operating time in laparoscopic anterior resection of rectal cancer

**DOI:** 10.1186/1477-7819-12-44

**Published:** 2014-02-25

**Authors:** Chu Wang, Yi Xiao, Huizhong Qiu, Jie Yao, Weidong Pan

**Affiliations:** 1 Department of General Surgery, Peking Union Medical College Hospital (PUMCH), Chinese Academy of Medical Sciences and Peking Union Medical College (CAMS & PUMC), Shuaifuyuan 1, Dongcheng District, Beijing 100730, China; 2 School of Biological Science and Medical Engineering, Beihang University, Xueyuan Road 37, Haidian District, Beijing 100191, China; 3 Department of Radiology, Peking Union Medical College Hospital (PUMCH), Chinese Academy of Medical Sciences and Peking Union Medical College (CAMS & PUMC), Beijing 100730, China

**Keywords:** Pelvimetry, Laparoscopic surgery, Rectal cancer

## Abstract

**Background:**

The objective of this study is to clarify the relationship between demographic and surgical factors and operating time, and thus operative difficulty, in patients undergoing laparoscopic anterior resection for mid-low rectal cancer, since different studies have derived different results.

**Methods:**

The records of patients with mid-low rectal cancer who underwent laparoscopic anterior resection were retrospectively studied. Demographic data, tumor characteristics, and pelvimetry measurements were collected and analyzed with respect to operating time, using correlation coefficient analysis, principle component analysis, and linear regression.

**Results:**

A total of 14 patients (10 males, 4 females; 65.50 ± 7.12 years of age) were included. Demographic and tumor characteristics not correlated with operating time. Body mass index (BMI) (*P* = 0.001); interacetabular distance (IA) (*P* = 0.001); anatomical transverse distance (IP) (*P* = 0.008); interischial distance (IS) (*P* = 0.002); intertuberous distance (IT) (*P* = 0.005); distance between the coccyx and symphysis (CoSy) (*P* = 0.013); and the angle of the lower border of the symphysis pubis, upper border of symphysis pubis, and sacral promontory (angle 5) (*P* = 0.004) were significantly associated with operating time. The equation was:

$$\begin{array}{l} \mathrm{operating}\;\mathrm{time}=\left(0.653\times \mathrm{BMI}\right)+\left(0.818\times \mathrm{angle}5\right)-\left(0.404\times \mathrm{IA}\right)\\ -\left(0.380\times \mathrm{IP}\right)-\left(0.512\times \mathrm{IS}\right)-\left(0.405\times \mathrm{IT}\right)-\left(0.570\times \mathrm{CoSy}\right)+330.8. \end{array}$$

**Conclusions:**

Transverse diameters of the pelvis, BMI, angle 5, and CoSy played the most important role in affecting operating time. The equation can be a very useful tool for preoperative assessment.

## Background

Laparoscopic anterior resection for mid-low rectal cancer (LRC) has now been widely accepted for its safety and for less blood loss and trauma than conventional open surgery, and the outcome of laparoscopic surgery has been demonstrated to be equivalent to that of open surgery [1,2]. However, LRC procedures need to be done within the pelvis, which leads to inherent technical operative difficulties.

Preoperative assessment of potential operative difficulties is quite important in preparation for LRC. For analyzing the factors affecting operative difficulty, operating time is typically chosen as the primary measure of difficulty as it is objective, and is well suited to a relatively small sample size in which some operative complications may not occur [3-6], and pelvimetry and tumor characteristics are expected to be reasonable. Some factors that have been shown to be associated with operative time and hence difficulty are high body mass index (BMI), narrow pelvic outlet, tumors closer to the anal verge, tumor T stage, previous abdominal surgery, and preoperative radiotherapy [3,4,6-9].

However, results derived from different studies are not identical [6,8,10,11], leading to difficulty in preoperative assessment. This may be because of sampling error, different techniques of measurement, or different definitions of difficulties and subgroups of independent variables. The purpose of this study was to further clarify the relationship between demographic and surgical factors and operating time, and thus operative difficulty, in patients undergoing LRC.

## Methods

### Patients and pelvimetry

Since LRC involves only the laparoscopic techniques and no stoma is required, it is suitable for assessing the relationship between operating time and affecting factors. A retrospective correlational study was performed in LRC patients. Due to the retrospective nature of the study, informed consent was waived. The study was approved by the Ethics Committee of our hospital.

Inclusion criteria were:

1) received LRC by one of two colorectal specialists (Professors H.Z. Qiu and Y. Xiao, who have each been performing laparoscopic operations for approximately 150 cases per year for more than 6 years) at our center from July 2012 to December 2012;

2) rectal adenocarcinoma confirmed by colonoscopy before surgery; and

3) distance of the tumor from anal verge ≤10 cm.

Exclusion criteria were:

1) distance of the tumor from anal verge >10 cm;

2) emergency surgery;

3) total mesorectal excision was not performed; or

4) other procedure was performed in addition to LRC, such as laparoscopic cholecystectomy.

**Figure 1:**
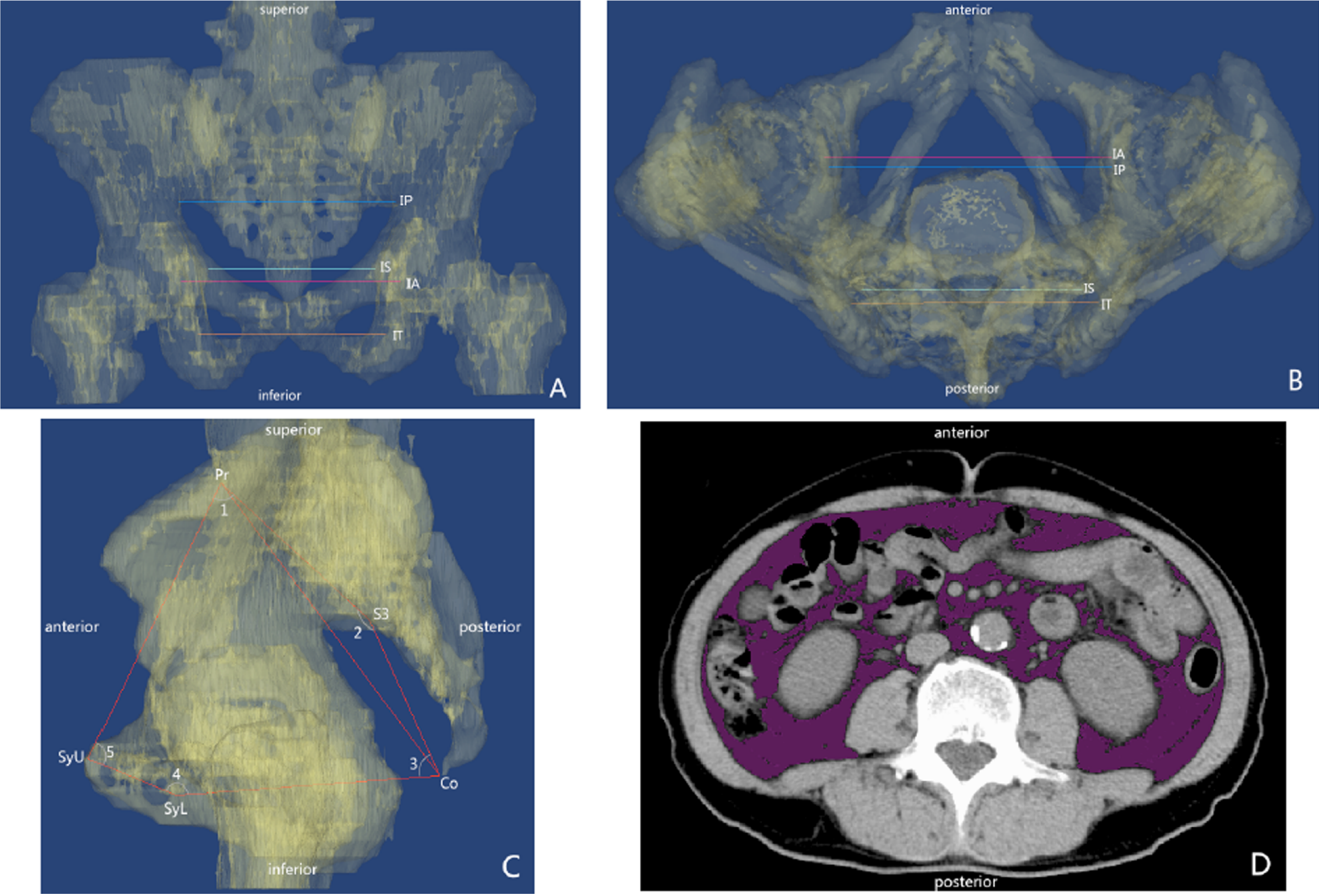
**Pelvimetry using Materialise’s Interactive Medical Image Control System. A) **Anterior-posterior transparent view of the pelvis; IP, anatomical transverse distance; IS, interischial distance; IA, interacetabular distance; IT, intertuberous distance. **B)** Superior-inferior transparent view of the pelvis. IP, anatomical transverse distance; IS, interischial distance; IA, interacetabular distance; IT, intertuberous distance. **C)** Lateral transparent view of the pelvis. Pr, promontory; S3, the middle point of the third sacrum; Co, coccyx; SyU, the upper border of symphysis pubis; SyL, the lower border of symphysis pubis; 1, angle 1; 2, angle 2; 3, angle 3; 4, angle 4; 5, angle 5. **D)** The purple shows the area of visceral adipose tissue at the single level of the umbilicus.

Preoperative data, including demographic characteristics, history of preoperative neoadjuvant chemoradiotherapy (NACR), history of lower abdominal surgery, BMI, and distance of the tumor from the anal verge (Td), were reviewed from the patients’ medical records. Postoperative pathological results were used to provide a precise description of the tumors (diameter and T stage). Abdominopelvic computed tomographic (CT) images were imported into Materialise’s Interactive Medical Image Control System (Mimics, version 10.01, Materialise, Belgium) [12]. Pelvimetry, as previous described [3,5], was measured twice via Mimics by one of our team (J. Yao), who was blinded to the clinical and histological outcomes of the cases studied. The mean of those data were calculated for further analysis. Operating time (opT) was collected from the medical records. This study was approved by the Ethic Committee of Peking Union Medical College. The pelvic variables measured are shown in Table 1 and Figure 1.

**Table 1:** Measurements of the pelvimetry and visceral fat area

**Measurement**	**Definition**
**Transverse measurements**	
Interacetabular distance (IA)	Distance between the most inner points of right and left femoral heads
Anatomical transverse distance (IP)	Distance between the most outer points of right and left iliopectineal lines
Interischial distance (IS)	Distance between the right and left ischial spines
Intertuberous distance (IT)	Distance between the most inner points of right and left ischial tuberosities
**Sagittal measurements**	
Pubis to promontory (SyPr)	Distance from the upper border of symphysis pubis to sacral promontory
Sacrum to promontory (S3Pr)	Distance from the middle point of the third sacrum to sacral promontory
Sacrum to coccyx (S3Co)	Distance from the middle point of the third sacrum to the tip of the coccyx
Promontory to coccyx (PrCo)	Distance from the sacral promontory to the tip of coccyx
Sacral depth (ScDep)	Distance from the deepest point of sacrum to the promontory-coccyx line
Pubis to coccyx (CoSy)	Distance from the lower border of symphysis pubis to the tip of coccyx
Length of symphysis pubis (SyLn)	Length of symphysis pubis
**Angles**	
Angle 1	The angle of the upper border of symphysis pubis to sacral promontory to the middle point of the third sacrum
Angle 2	The angle of sacral promontory to the middle point of the third sacrum to the tip of coccyx
Angle 3	The angle of the middle point of the third sacrum to the tip of coccyx to the lower border of symphysis pubis
Angle 4	The angle of the tip of coccyx to the lower border of symphysis pubis to the upper border of symphysis pubis
Angle 5	The angle of the lower border of symphysis pubis to the upper border of symphysis pubis to the sacral promontory
**Visceral fat area (VFA)**	The area of visceral adipose tissue at the single level of the umbilicus

### Statistical analysis

Statistical analyses were performed using SPSS Statistics 17.0 software (SPSS Inc., Chicago, IL, USA). Descriptive analyses were used to characterize the study population. Normally distributed data were examined using the Shapiro-Wilk test. For continuous data with a normal distribution, Pearson’s correlation coefficient was used to determine the relationships between factors and operating time; otherwise Spearman’s ranking correlation coefficient was applied. Univariate analysis was used to assess the relationship between gender, tumor T stage, history of lower abdominal surgery, and NACR with operating time. Linear regression with collinearity diagnostics was performed for data correlated with operating time. If collinearity existed, principle components analysis was applied to further explore the internal relationship between factors and operating time. A *P* value of less than 0.05 was considered significant.

## Results

A total of 14 patients (10 males, 4 females) with a mean age of 65.50 ± 7.12 years were included in this retrospective study (Table 2). No patient had a history of a previous abdominal surgery. One patient was stage Tis, One patient was stage T1, three patients were stage T2, eight patients were stage T3 and one patient was stage T4. The mean opT of LRC was 171.43 ± 48.18 minutes [see Additional file 1: Table S1].

**Table 2:** Statistical results of factors affecting operating time

**Variables (unit)**	**Description mean ± SD (minimal, maximal) or median (interquartile range)**	**Correlation with operating time**	
		**r or F**	***P***
Operating time (min)	171.43 ± 48.18 (100, 260)	-	-
Age (years old)	65.50 ± 7.12 (54, 76)	0.174	0.553
Gender^a^	-	3.102	0.138
NACR^a^	-	0.937	0.377
BMI^b^	22.88 (21.94, 24.57)	0.779	0.001
VFA (cm^2^)	106.83 ± 47.19 (4.52, 162.07)	0.296	0.304
diameter of tumor (cm)	2.91 ± 1.32 (1, 5)	-0.225	0.439
T stage of tumor^a^	-	0.736	0.606
Td (cm)^b^	8.00 (6.75, 10.0)	-0.328	0.252
IA (mm)	128.76 ± 12.22 (111.36, 158.92)	-0.771	0.001
IS (mm)	104.65 ± 15.47 (84.55, 131.36)	-0.760	0.002
IT (mm)	101.24 ± 17.89 (79.71, 134.63)	-0.704	0.005
IP (mm)	133.97 ± 16.30 (113.21, 170.18)	-0.676	0.008
SyPr (mm)	120.74 ± 12.78 (99.47, 145.94)	0.066	0.822
S3Pr (mm)	82.54 ± 9.61 (63.77, 99.28)	-0.063	0.831
S3Co (mm)	69.12 ± 8.94 (56.11, 87.20)	0.494	0.072
PrCo (mm)	137.82 ± 14.24 (117.77, 175.47)	0.304	0.291
ScDep (mm)	32.99 ± 4.54 (22.16, 41.45)	0.077	0.793
CoSy (mm)	103.99 ± 11.33 (86.17, 121.51)	-0.646	0.013
SyLn (mm)	40.27 ± 4.80 (30.73, 47.50)	-0.032	0.913
Angle 1 (°)	84.86 ± 12.16 (62.99, 109.48)	-0.430	0.125
Angle 2 (°)	130.78 ± 8.47 (111.80, 142.61)	0.137	0.640
Angle 3 (°)	92.22 ± 6.46 (77.98, 103.87)	0.146	0.619
Angle 4 (°)	131.79 ± 7.96 (117.39, 146.16)	-0.504	0.066
Angle 5 (°)	100.17 ± 9.99 (84.11, 123.25)	0.716	0.004

^a^Univariate analysis was applied.

^b^Spearman’s ranking correlation coefficient was applied. For the rest of the variables, Pearson’s correlation coefficient was applied.

BMI: body mass index; CoSy: distance from the lower border of symphysis pubis to the tip of coccyx; IA: interacetabular distance; IP: anatomical transverse distance; IS: interischial distance; IT: intertuberous distance; NACR: neoadjuvant chemoradiotherapy; PrCo: distance from the sacral promontory to the tip of coccyx; ScDep: distance from the deepest point of sacrum to the promontory-coccyx line; SyLn: Length of symphysis pubis; SyPr: distance from the upper border of symphysis pubis to sacral promontory; S3Co: distance from the middle point of the third sacrum to the tip of the coccyx; S3Pr: distance from the middle point of the third sacrum to sacral promontory; Td: tumor distance from the anal verge; VFA: visceral fat area.

Table 2 shows the correlations that were found between BMI, IA, IP, IS, IT, CoSy, and angle 5 with opT, while other factors including age, gender, NACR, VFA, tumor diameter, tumor T stage, Td, SyPr, S3Pr, S3Co, PrCo, ScDep, SyLn, angle 1, angle 2, angle 3, and angle 4, were not correlated with operating time.

Linear regression with collinearity diagnostics showed collinearity existed within the correlated factors (eigenvalue = 0.008, condition index = 25.322). Dimension reduction using principle component analysis was applied after the variables were standardized (Z-score). If more than 85% of the variance can be explained by several components (principle components), the principle components can be regarded as the major factors, while the other components can be ignored. Principle component analysis showed that 89.06% of the total variance could be explained by two principle components [see Additional file 2: Table S2], so two principle components (Z1 and Z2) were calculated, and linear regression analysis was applied:

Z-score–opT=-0.342×Z1+0.097×Z2.

After return operation, the final equation could be obtained as:

$$\begin{array}{l} \mathrm{opT}=\left(0.653\times \mathrm{BMI}\right)+\left(0.818\times \text{angle}5\right)-\left(0.404\times \mathrm{IA}\right)\\ \;-\left(0.380\times \mathrm{IP}\right)-\left(0.512\times \mathrm{IS}\right)-\left(0.405\times \mathrm{IT}\right)\\ \;-\left(0.570\times \mathrm{CoSy}\right)+330.8. \end{array}$$

## Discussion

Factors affecting the operating time of LRC have drawn attention in recent years, and a great deal of effort has been given to validating the relationship between factors and operating time [3,6,8,10,11,13,14]. Tumor diameter, BMI, operator experience, tumor distance from the anal verge, tumor depth, pelvic outlet, gender, and VFA have been demonstrated to be related to operating time [6,8,10,11]. However, different studies have provided different conclusions, leading to confusion, and no discussions on how the various factors correlate with each other and the operating time were presented. This might be because of different definitions of subgroups and difficulties, or sampling error. In this study, we focused on the establishment of a more precise description of the relationship between factors and operating time. According to previous studies and our own experience, we assumed that pelvimetry, in addition to demographic characteristics, was quite important in LRC. Two-dimensional magnetic resonance imaging (MRI) and X-ray images have been used to measure pelvimetry [3,4]; however, if the patient is not positioned symmetrically, deviation of the measurements will result. In order to describe the pelvis more precisely, three-dimensional measurements were adopted using Mimics in this study (Figure 1).

The equation we obtained showed the internal relationship between the various factors studied. Based on the equation, BMI, angle 5, transverse diameters of the pelvis, and CoSy were related to operating time. However none of the factors was the dependent factor affecting operating time. Thus, the frame of the pelvis should be considered as a whole. BMI and angle 5 have positive effects on operating time, while transverse diameters (IA, IP, IS and IT) and CoSy have negative effects on opT. According to the coefficients in the equation, a wider pelvis, especially with a ‘bigger’ pelvic floor, could reduce the difficulty of the operation, while increased angle 5 may increase the operating time for the step of anterior dissection, which needs further verification via step-by-step timing. Besides the anatomical factors, BMI, which could reflect the soft tissue volume in the pelvis, was also very important in affecting operating time.

This equation can be used as a very useful tool for preoperative assessment of patients undergoing LRC. If calculated or predicted operating time is more than a given time, the technique might not be suitable for junior specialists without extensive training. Further research should be performed to identify the given time.

### Advantages and limitations

The advantage is that we used three-dimensional measurements to obtain pelvimetry data, which is much more accurate than that of two-dimensional measurements. We described the relationship between factors and operating time more accurate by the equation, which provides the internal relationship among the factors. The small number of patients is the major limitation of the study. In addition, the timings for different steps of the procedure were not been recorded, which lead to step-by-step analysis of the procedures impossible. We recognized the limitations of the retrospective study; nevertheless, we believe that this study provides important information for further research.

## Conclusions

No demographic datum and any measurement of pelvimetry could not be identified as an independent predictor for operating time in our study. Instead, transverse diameters of the pelvis, BMI, angle 5, and CoSy played the most important role in affecting operating time. The equation reveals the internal relationship among the factors, and it can be a very useful tool for preoperative assessment.

## Abbreviations

BMI: body mass index; CoSy: distance from the lower border of symphysis pubis to the tip of coccyx; CT: computed tomography; IA: interacetabular distance; IP: anatomical transverse distance; IS: interischial distance; IT: intertuberous distance; LRC: Laparoscopic anterior resection for mid-low rectal cancer; Mimics: Materialise’s Interactive Medical Image Control System; MRI: magnetic resonance imaging; NACR: neoadjuvant chemoradiotherapy; PrCo: distance from the sacral promontory to the tip of coccyx; ScDep: distance from the deepest point of sacrum to the promontory-coccyx line; SyLn: Length of symphysis pubis; SyPr: distance from the upper border of symphysis pubis to sacral promontory; S3Co: distance from the middle point of the third sacrum to the tip of the coccyx; S3Pr: distance from the middle point of the third sacrum to sacral promontory; Td: tumor distance from the anal verge; VFA: visceral fat area.

## Competing interests

The authors declare that they have no competing interests.

## Authors’ contributions

CW collected and analyzed the medical data. JY measured the pelvimetry. HZQ and YX helped analyze the medical records. WDP collected the computed tomographic images. All authors read and approved the final manuscript.

## Supplementary Material

Additional file 1: Table S1 Original demographic data of the patients and tumor information. Click here for file

Additional file 2: Table S2 Dimension reduction analysis. Click here for file
