# Supplementary material for: Factors affecting operating time in laparoscopic anterior resection of rectal cancer
Source: World J Surg Oncol. 2014 Feb 25;12:44. doi: 10.1186/1477-7819-12-44 (PMC3941695; doi:10.1186/1477-7819-12-44)
Supplement: Additional file 1: Table S1 — Original demographic data of the patients and tumor information. [file 1477-7819-12-44-S1.doc]

Supplementary table 1 : Dimension reduction analysis

| **Total Variance Explained** | | | | | | | |
| --- | --- | --- | --- | --- | --- | --- | --- |
| Component | | Initial Eigenvalues^a^ | | | Extraction Sums of Squared Loadings | | |
|  |  | Total | % of Variance | Cumulative % | Total | % of Variance | Cumulative % |
| Raw | 1 | 5.382 | 76.884 | 76.884 | 5.382 | 76.884 | 76.884 |
|  | 2 | 0.852 | 12.176 | 89.060 | 0.852 | 12.176 | 89.060 |
|  | 3 | 0.306 | 4.370 | 93.430 |  |  |  |
|  | 4 | 0.250 | 3.577 | 97.007 |  |  |  |
|  | 5 | 0.137 | 1.960 | 98.967 |  |  |  |
|  | 6 | 0.064 | 0.913 | 99.880 |  |  |  |
|  | 7 | 0.008 | 0.120 | 100.000 |  |  |  |
| Rescaled | 1 | 5.382 | 76.884 | 76.884 | 5.382 | 76.884 | 76.884 |
|  | 2 | 0.852 | 12.176 | 89.060 | 0.852 | 12.176 | 89.060 |
|  | 3 | 0.306 | 4.370 | 93.430 |  |  |  |
|  | 4 | 0.250 | 3.577 | 97.007 |  |  |  |
|  | 5 | 0.137 | 1.960 | 98.967 |  |  |  |
|  | 6 | 0.064 | 0.913 | 99.880 |  |  |  |
|  | 7 | 0.008 | 0.120 | 100.000 |  |  |  |
| Extraction Method: Principal Component Analysis. | | | | | | | |
| a. When analyzing a covariance matrix, the initial eigenvalues are the same across the raw and rescaled solution. | | | | | | | |
