# Supplementary material for: Factors affecting operating time in laparoscopic anterior resection of rectal cancer
Source: World J Surg Oncol. 2014 Feb 25;12:44. doi: 10.1186/1477-7819-12-44 (PMC3941695; doi:10.1186/1477-7819-12-44)
Supplement: Additional file 2: Table S2 — Dimension reduction analysis. [file 1477-7819-12-44-S2.doc]

Supplementary table 2： Original demographic data of the patients and tumor information

| Patient’s No. | Age (years) | Gender | HisLoAbSur^a^ | NACR^b^ | Height  (cm) | Weight  (kg) | Size of tumor  (cm) | Td^c^  (cm) | T stage of tumor^d^ |
| --- | --- | --- | --- | --- | --- | --- | --- | --- | --- |
| 1 | 54 | Male | No | No | 173 | 68 | 4 | 7 | 3 |
| 2 | 59 | Female | No | No | 162 | 47 | 5 | 8 | 2 |
| 3 | 72 | Male | No | No | 170 | 65 | 5 | 8 | 4 |
| 4 | 61 | Male | No | No | 178 | 110 | 2.3 | 6 | 1 |
| 5 | 73 | Male | No | No | 170 | 60 | 3 | 8 | tis |
| 6 | 72 | Male | No | No | 164 | 76 | 3.4 | 10 | 3 |
| 7 | 60 | Male | No | Yes | 178 | 67 | 1.2 | 7 | 3 |
| 8 | 76 | Female | No | Yes | 160 | 65 | 2 | 7 | 2 |
| 9 | 73 | Female | No | No | 150 | 50 | 3.5 | 10 | 3 |
| 10 | 67 | Male | No | Yes | 185 | 82 | 3 | 6 | 3 |
| 11 | 63 | Male | No | No | 169 | 68 | 4 | 10 | 3 |
| 12 | 64 | Male | No | Yes | 178 | 77 | 1 | 6 | 3 |
| 13 | 55 | Female | No | No | 160 | 59 | 2 | 9 | 3 |
| 14 | 68 | Male | No | No | 175 | 68 | 1.4 | 10 | 2 |

Note：a. HisLoAbSur = History of lower abdominal surgery

b. NACR = neoadjuvant chemoradiotherapy

c. Td = distance of the tumor from the anal verge

d. The staging was based on the NCCN Guidelines Version 3.2011 Staging Rectal Cancer
